# Vascular Dysfunction in Diabetes and Obesity: Focus on TRP Channels

**DOI:** 10.3389/fphys.2021.645109

**Published:** 2021-02-26

**Authors:** Raiana dos Anjos Moraes, R. Clinton Webb, Darízy Flávia Silva

**Affiliations:** ^1^Laboratory of Cardiovascular Physiology and Pharmacology, Institute of Health Sciences, Federal University of Bahia, Salvador, Brazil; ^2^Postgraduate Course in Biotechnology in Health and Investigative Medicine, Gonçalo Moniz Institute, Oswaldo Cruz Foundation (FIOCRUZ), Salvador, Brazil; ^3^Department of Cell Biology and Anatomy and Cardiovascular Translational Research Center, University of South Carolina, Columbia, SC, United States

**Keywords:** TRP channels, vascular dysfunction, diabetes, obesity, TRPC, TRPM, TRPML, TRPV

## Abstract

Transient receptor potential (TRP) superfamily consists of a diverse group of non-selective cation channels that has a wide tissue distribution and is involved in many physiological processes including sensory perception, secretion of hormones, vasoconstriction/vasorelaxation, and cell cycle modulation. In the blood vessels, TRP channels are present in endothelial cells, vascular smooth muscle cells, perivascular adipose tissue (PVAT) and perivascular sensory nerves, and these channels have been implicated in the regulation of vascular tone, vascular cell proliferation, vascular wall permeability and angiogenesis. Additionally, dysfunction of TRP channels is associated with cardiometabolic diseases, such as diabetes and obesity. Unfortunately, the prevalence of diabetes and obesity is rising worldwide, becoming an important public health problems. These conditions have been associated, highlighting that obesity is a risk factor for type 2 diabetes. As well, both cardiometabolic diseases have been linked to a common disorder, vascular dysfunction. In this review, we briefly consider general aspects of TRP channels, and we focus the attention on TRPC (canonical or classical), TRPV (vanilloid), TRPM (melastatin), and TRPML (mucolipin), which were shown to be involved in vascular alterations of diabetes and obesity or are potentially linked to vascular dysfunction. Therefore, elucidation of the functional and molecular mechanisms underlying the role of TRP channels in vascular dysfunction in diabetes and obesity is important for the prevention of vascular complications and end-organ damage, providing a further therapeutic target in the treatment of these metabolic diseases.

## Introduction

Diabetes mellitus and obesity are characterized by systemic biochemical and biological abnormalities, including metabolic disturbances, increased oxidative stress (Pandey et al., 2010; Fülöp et al., 2014; D’souza et al., 2016), and elevated circulating levels of inflammatory markers (Panagiotakos et al., 2005; Taha et al., 2019). Obesity is a condition related to disproportionate body weight for height with an excessive accumulation of adipose tissue (González-Muniesa et al., 2017). Moreover, obesity represents the strongest risk factor for type 2 diabetes (Censin et al., 2019), and it is a common comorbidity among type 2 diabetics (Fajarini and Sartika, 2019). On the other hand, diabetes mellitus can be classified into many subtypes, which can be characterized and identified by the presence of hyperglycemia (World Health Organization, 2019).

Unfortunately, the prevalence of these cardiometabolic disorders has been increasing worldwide (Abarca-Gómez et al., 2017; International Diabetes Federation, 2019). Additionally, it is evident that diabetes and obesity are related with enhanced cardiovascular risk (Ärnlöv et al., 2010; Einarson et al., 2018). Moreover, these cardiometabolic disorders have been linked to a common condition: vascular dysfunction (Schofield et al., 2002; Oltman et al., 2006; Sivitz et al., 2007; Farb et al., 2014). For instance, diabetic and obese individuals can both be affected by an impaired functional endothelium (Steinberg et al., 1996; Doupis et al., 2011) and/or increased vasoconstriction (Hogikyan et al., 1999; Cardillo et al., 2002; Weil et al., 2011; Schinzari et al., 2015), thus leading to vascular complications. Currently, there are a large number of studies that have described the mechanisms of vascular dysfunction, which involve altered transient receptor potential (TRP) channels expression and/or activity, a common event observed in hypertension (Mathar et al., 2010; Alves-Lopes et al., 2020), atherosclerosis (Wei et al., 2013; Zhao et al., 2016), pulmonary hypertension (Yu et al., 2004; Yang et al., 2012) and pulmonary edema (Jian et al., 2008; Thorneloe et al., 2012). Therefore, these channels could provide additional targets for treatment of these vascular diseases. Furthermore, TRP channels are involved in diabetic (Evans et al., 2009; Lu et al., 2014; Monaghan et al., 2015; Zhang et al., 2015) and obesity-related (Zhang et al., 2007; Lee et al., 2015; Sun et al., 2019; Ottolini et al., 2020) diseases. TRP superfamily consists of a diverse group of non-selective cation channels that is divided into six subfamilies in mammals, which are classified as: canonical or classical (TRPC), vanilloid (TRPV), melastatin (TRPM), ankyrin (TRPA), mucolipin (TRPML), and polycystin (TRPP) (Montell, 2005; Ramsey et al., 2006). Additionally, this superfamily is distributed throughout a variety of body tissues, such as blood vessels (Mita et al., 2010; Gao et al., 2020), heart (Andrei et al., 2016), brain (Tóth et al., 2005) and bladder (Yu et al., 2011), among others.

In this context, the correlation between TRP channels, diabetes and obesity have continued to attract growing attention. In this review, we briefly consider general features of TRP channels and focus on TRPC, TRPV, TRPM, and TRPML, which have been shown to be potential involved in the vascular dysfunction of diabetes and obesity.

## Overview on Diabetes and Obesity

Globally, an estimated 463 million individuals were affected by diabetes in 2019. The International Diabetes Federation estimates that there will be 578 million adults with diabetes by 2030, and 700 million by 2045. Unfortunately, the global high prevalence of diabetes continues to increase, with no indications of stabilizing (International Diabetes Federation, 2019).

Similarly, the prevalence of obesity is rising in the world. The global number of girls with obesity rose from 5 million in 1975 to 50 million, and the number of boys increased from 6 million in 1975 to 74 million in 2016. As well, the number of adult women with obesity rose from 69 million in 1975 to 390 million, and the number of men grew from 31 million in 1975 to 281 million in 2016 (Abarca-Gómez et al., 2017). In addition, from 2017 to 2018, the prevalence of obesity in the United States was 42.4%, and the prevalence of severe obesity was 9.2% among adults (Hales et al., 2020). The study by Sonmez et al. (2019) demonstrated a high prevalence of obesity in patients with type 2 diabetes, where only 10% of patients with type 2 diabetes had normal body mass indexes (BMI), while the remaining patients were either overweight (31%) or obese (59%).

Worldwide, an estimated 41 million people died of non-communicable diseases (NCDs) in 2016, corresponding to 71% of all deaths. Cardiovascular diseases (17.9 million deaths), cancer (9.0 million deaths), chronic respiratory diseases (3.8 million deaths), and diabetes (1.6 million deaths) were the four greatest contributors of NCDs related deaths. The increasing mortality rates in diabetic cases are related with the rising prevalence of obesity and other factors (World Health Organization, 2020).

Obesity has been linked to increased risk of various chronic diseases, including type 2 diabetes, coronary artery disease, stroke, and fatty liver (Censin et al., 2019). Moreover, diabetes is strongly related with nephropathy, retinopathy, neuropathy (Nathan et al., 2015; Garofolo et al., 2019), and erectile dysfunction (Kouidrat et al., 2017; Carrillo-Larco et al., 2018). These diseases are associated with increased risk of cardiovascular disease, elevated mortality, low quality of life (Silveira et al., 2020), and increased financial burden to health care systems. Therefore, diabetes and obesity are considered important global public health concerns (Hex et al., 2012).

## Vascular Complications of Diabetes and Obesity

**FIGURE 1:**
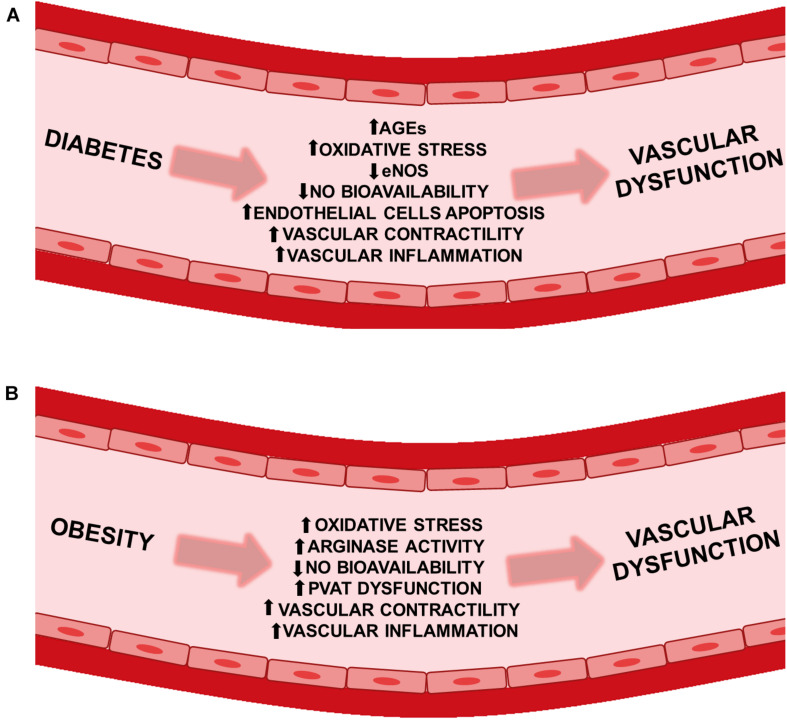
Vascular dysfunction in diabetes and obesity. Pathophysiological factors leading to vascular dysfunction in **(A)** diabetic and **(B)** obese patients. AGEs, advanced glycation end products; eNOS, endothelial nitric oxide synthase; NO, nitric oxide; PVAT, perivascular adipose tissue.

Type 2 diabetes is associated with the onset of microvascular complications, such as nephropathy, retinopathy and neuropathy, as well as macrovascular complications, including coronary artery disease and cerebrovascular disease (Litwak et al., 2013; Kosiborod et al., 2018). A study by van Wijngaarden et al. (2017) demonstrated that the greater and more prolonged exposure to hyperglycemia, enhances the risk of both microvascular and macrovascular complications in patients with type 2 diabetes (van Wijngaarden et al., 2017). Comparably, intensive glucose control significantly reduced adverse outcomes due to major macrovascular or microvascular events (Patel et al., 2008). Moreover, obesity and type 2 diabetes mellitus in adolescents, predispose this group to higher vascular disease risk (Ryder et al., 2020). As well, overweight and obese individuals had an increased risk for major cardiovascular events, such as: myocardial infarction, stroke, and heart failure (Ärnlöv et al., 2010). Additionally, there are several factors that contribute to the vascular dysfunction associated with diabetes. Chronic hyperglycemia has been shown to impair endothelium-dependent vasodilatation in diabetes (Mäkimattila et al., 1996). Elevated advanced glycation end products (AGEs) has been shown to cause endothelial dysfunction (Xu B. et al., 2003; Ren et al., 2017). Similarly, increased oxidative stress can reduce nitric oxide (NO) bioavailability (Nassar et al., 2002; Cho et al., 2013), while augmented peroxynitrite may inactivate endothelial nitric oxide synthase (eNOS) (Chen et al., 2010; Cassuto et al., 2014). As well, augmented vascular contractility (Xie et al., 2006; Matsumoto et al., 2014; Lubomirov et al., 2019), increased vascular inflammation (Zhang et al., 2008; Ku and Bae, 2016), and stimulated endothelial cells apoptosis (Sheu et al., 2005, Sheu et al., 2008) can cooperate to cause vascular dysfunction (Figure 1A). There are key processes in obesity which collaborate and lead to impairment of vascular function. These processes include enhanced vascular contractility (Boustany-Kari et al., 2007; Weil et al., 2011), augmented sympathetic control of vasoconstriction (Haddock and Hill, 2011), elevated oxidative stress (La Favor et al., 2016), increased peroxynitrite (Mason et al., 2011; Gamez-Mendez et al., 2015), perivascular adipose tissue (PVAT) dysfunction (Ma et al., 2010; Bussey et al., 2016), increased arginase activity (which can reduce L-arginine and NO bioavailability) (Johnson et al., 2015; Bhatta et al., 2017), and increased vascular inflammation (Yao et al., 2017; Figure 1B). Both diabetes and obesity share common mechanisms that result in vascular injury. Thus, elucidation of the mechanisms underlying vascular dysfunction in these cardiometabolic diseases is essential to provide additional therapeutic targets in the prevention and treatment of these cardiometabolic diseases. Interestingly, alterations in TRPs channel expression or/and function may contribute to these pathological conditions, making these channels promising therapeutic targets.

## TRP Channels

The TRP superfamily was originally discovered in the study on *Drosophila melanogaster*, where in response to bright light, *Drosophila* mutants behaved as if they were blind, while wild-type flies maintained oriented toward visual cues. Thus, in the mutated eye, the light-response was transient during sustained light (Cosens and Manning, 1969). This mutant was known as TRP due to the transient response to prolonged intense lights, performed by Minke and colleagues (Minke et al., 1975). Following these reports, the molecular characterization of the *Drosophila* TRP gene was described (Montell and Rubin, 1989).

In addition, a common feature in the TRP superfamily is its tetrameric structure, where each subunit is constituted by six transmembrane segments, a pore-forming region between the segments S5–S6 and cytoplasmic amino and carboxyl termini (For general explanation, see reviews by: Earley and Brayden, 2015; Hof et al., 2019). Mammalian genomes encode 28 distinct TRP protein subunits, and this superfamily is divided into six subfamilies, based on amino acid sequence homology and include: TRPC (Wes et al., 1995; Liu et al., 2008), TRPV (Caterina et al., 1997; Smith et al., 2002), TRPM (Tsavaler et al., 2001; Fujiwara and Minor, 2008), TRPA (Story et al., 2003; Cvetkov et al., 2011), TRPP (Mochizuki et al., 1996; Giamarchi et al., 2010), and TRPML (Sun et al., 2000; Zeevi et al., 2010).

The TRP superfamily consists of a diverse group of cation channels, where most of the channels are non-selective and permeable to Ca^2+^ (Gonzalez-Perrett et al., 2001; Feng et al., 2014; Sierra-Valdez et al., 2018). These channels have been shown to be involved in many physiological processes, such as responses to painful stimuli (Caterina et al., 2000; Davis et al., 2000), repletion of intracellular calcium stores (Rosado et al., 2002), vasoconstriction/vasorelaxation (Freichel et al., 2001; Dietrich et al., 2005), secretion of hormones (Togashi et al., 2006; Cheng et al., 2007), cell cycle modulation (Lee et al., 2011; Tajeddine and Gailly, 2012), sensory perception (Kichko et al., 2018) and others. This superfamily displays a variety of activation mechanisms, such as ligand binding (Janssens et al., 2016), temperature (McKemy et al., 2002), endogenous chemical mediators (Beck et al., 2006), voltage (Matta and Ahern, 2007), G protein-coupled receptors (Boulay et al., 1997), and tyrosine kinase receptors (Xu H. et al., 2003; Vazquez et al., 2004), among other stimuli.

In blood vessels, TRP channels are present in endothelial cells (Ching et al., 2011), vascular smooth muscle cell (VSMC) (Johnson et al., 2009), PVAT (Sukumar et al., 2012), perivascular sensory nerves (Zygmunt et al., 1999), and pericytes (Tóth et al., 2005), and these channels have been implicated in the regulation of vascular tone (Pórszász et al., 2002; Qian et al., 2007; Earley et al., 2009), vascular cell proliferation (Zhang et al., 2018), vascular wall permeability (Tiruppathi et al., 2002; Paria et al., 2004), and angiogenesis (Hamdollah Zadeh et al., 2008; Ge et al., 2009). Additionally, there are a large number of studies describing the involvement of TRP proteins in various pathophysiological conditions. We focus on altered expression and/or activity of the TRPC, TRPV, TRPM, and TRPML channels, contributing to vascular dysfunction in obese and diabetic conditions or are potentially associated to vascular alterations.

## TRP Channels Involved in Vascular Complications of Diabetes and Obesity

### The Role of TRPC in the Vasculature Under Diabetic and Obese Conditions

The TRPC subfamily consists of seven proteins, known as TRPC1 to TRPC7 (see review of Clapham et al., 2001; Putney, 2005; Dietrich et al., 2010; Mederos y Schnitzler et al., 2018). TRPC channels can form homo- and heterotetramers (Hofmann et al., 2002; Strübing et al., 2003). Moreover, there is increasing evidence that TRPC channel members can form receptor-operated channels (ROC) (Soboloff et al., 2005; Peppiatt-Wildman et al., 2007; Tai et al., 2008; Inoue et al., 2009; Itsuki et al., 2014) and store-operated channels (SOC) (Groschner et al., 1998; Freichel et al., 2001; Xu and Beech, 2001; Xu et al., 2006; Shi et al., 2016).

TRPC1, TRPC3, TRPC4, TRPC5, and TRPC6 are expressed in VSMC (Wang et al., 2004; Evans et al., 2009; Inoue et al., 2009; Mita et al., 2010) and endothelial cells (Yip et al., 2004; Gao et al., 2012; Sundivakkam et al., 2012). TRPC channels are involved in the regulation of vascular tone through different signaling pathways. For example, activation of TRPC1 and TRPC3 channels in the VSMC can cause depolarization and vasoconstriction (Reading et al., 2005; Wölfle et al., 2010). Alternatively, TRPC1 channels can be associated with large-conductance Ca^2+^-activated K^+^ (BK_*Ca*_) channels in VSMC, indirectly activating cell hyperpolarization (Kwan et al., 2009). As well, TRPC1, TRPC3, and TRPC4 stimulation in endothelial cells can induce vasodilation through increases in endothelial Ca^2+^, with subsequent generation of NO (Freichel et al., 2001; Huang et al., 2011; Qu et al., 2017) and/or TRPC3 activation can induce endothelium-dependent hyperpolarization factor (EDHF)-mediated vasodilation (Kochukov et al., 2014). However, only a few studies have demonstrated the involvement of TRPC channels in the vasculature of diabetic animals and humans and no studies have investigated a role for TRPC in obesity.

Evans et al. (2009) showed that angiotensin-II (Ang-II)-induced Ca^2+^ influx was significantly enhanced in cultured aortic VSMC from Goto-Kakizaki (GK) rats, a model of type 2 diabetes, when compared with cells from Wistar-Kyoto (WKY) control rats. TRPC1 and TRPC5 protein expression were similar, while TRPC4 protein expression was significantly increased, and TRPC6 protein expression was significantly decreased in GK, compared with WKY values. In GK-VSMC, Ang-II-induced Ca^2+^ influx was more sensitive to the calcium influx inhibitors 2-aminoethoxydiphenyl borate (2-APB) and caffeine, which act through the inhibition of the inositol 1,4,5-trisphosphate receptor (IP_3_R). Since TRPC1 can be activated by an IP_3_R coupling mechanism, this result suggests a possible increased activation of mechanisms contributing to TRPC1 activity. The authors of this study proposed that the elevated calcium influx induced by Ang-II was due to the alteration of TRPC1/4/5 activity in diabetic rats (Evans et al., 2009). However, 2-APB and caffeine are non-selective inhibitors and therefore, the general absence of selective pharmacological tools for TRPC channels is a study limitation. Additionally, 2-APB and caffeine cannot be considered as specific reagents to evaluate TRPC1 activity. Therefore, the use of gene knockout or knockdown animals could offer a valuable alternative for studying specific functions of TRPC channels in the regulation of vascular tone in diabetic conditions. However, a limitation of this approach is that when one TRPC channel is downregulated or knocked out it may be compensated by other TRPCs, as evidenced by Dietrich et al. (2005). Therefore, these obstacles make difficult to draw correct conclusions about the role of TRPC channels on the obesity and diabetes.

A study by Chung and colleagues provided the first evidence that TRPC1, TRPC4 and TRPC6 messenger RNA (mRNA) and proteins are present in human saphenous vein, and their expression levels are modulated by type II diabetes. The authors demonstrated that cyclopiazonic acid (CPA)-induced contraction of the saphenous vein was greater in diabetic vessels than the non-diabetic, suggesting that the increased contractility in human diabetes could be partially due to the participation of Ca^2+^ entry through SOC. Additionally, TRPC channels may be involved in SOC. Although TRPC4 mRNA expression was elevated, protein levels were not significantly different when compared to non-diabetic vessels. TRPC1 and TRPC6 mRNA levels in diabetic conditions were similar to the control, however, protein expression was decreased in diabetic veins. Even though TPRC protein expression was diminished in the diabetic samples, the enhanced CPA-induced contraction in diabetic veins might be associated with increased TRPC activity, leading to higher capacitative Ca^2+^ entry (Chung et al., 2009).

Mita and colleagues demonstrated that TRPC1, TRPC3, and TRPC6 mRNAs and proteins were expressed in caudal arteries from Wistar rats. However, in addition to the expression of these TRPC channels, TRPC4 also was expressed at extremely low levels in GK rats. In addition, GK rats had a significant increase in protein expression of TRPC1 and TRPC6 channels or appearance of TRPC4 channel expression, but not TRPC3, compared with Wistar rats, which is associated with the reduction in cirazoline- or CPA-induced contractions in GK (Mita et al., 2010).

These authors demonstrated that TRPC channel expression levels and function are altered in diabetes (Table 1). However, there was a heterogeneity of findings among these studies, therefore these discrepancies may be explained by a number of factors, including: variations in the metabolic profile of the diabetic animals, distinct stages of diabetes, and the type of arteries and veins investigated. Nevertheless, the *in vivo* significance of these findings has not been shown. Additionally, the role of TRPCs in obesity should also be more completely explored in future studies.

**TABLE 1:** TRP channels involved in vascular complications of diabetes and obesity.

TRP channels involved in vascular complications of diabetes and obesity							
Channel	Diabetic and/or obesity model	Normal control	Tissue	Drug-induced vascular effect or other vascular investigations	mRNA in diabetic and/or obesity model	Protein in diabetic and/or obesity model	References
TRPC1	Goto-Kakizaki (GK) (Type 2 diabetes)	Wistar-Kyoto (WKY) rat	Cultured aortic vascular smooth muscle cell	Angiotensin-II-induced Ca^2+^ influx was enhanced in diabetic rat	Decrease	No change	Evans et al. (2009)
TRPC1	*Human Type II diabetic*	Human non-diabetic	Saphenous vein	Cyclopiazonic acid-induced Ca^2+^ influx was enhanced in diabetic patient	No change	Decrease	Chung et al. (2009)
TRPC1	GK rat	Wistar rats	Endothelium-denuded caudal artery smooth muscle strips	Cirazoline- or cyclopiazonic acid-induced Ca^2+^ influx was decreased in diabetic rat	–	Increase	Mita et al. (2010)
TRPC3	GK rat	WKY rat	Cultured aortic vascular smooth muscle cell	Angiotensin-II-induced Ca^2+^ influx was enhanced in diabetic rat	Undetectable	–	Evans et al. (2009)
TRPC3	GK rat	Wistar rat	Endothelium-denuded caudal artery smooth muscle strips	Cirazoline- or cyclopiazonic acid-induced Ca^2+^ influx was decreased in diabetic rat	–	No change	Mita et al. (2010)
TRPC4	GK rat	WKY rat	Cultured aortic vascular smooth muscle cell	Angiotensin-II-induced Ca^2+^ influx was enhanced in diabetic rat	No change	Increase	Evans et al. (2009)
TRPC4	Human diabetic	Human non-diabetic	Saphenous vein	Cyclopiazonic acid-induced Ca^2+^ influx was enhanced in diabetic patient	Increase	No change	Chung et al. (2009)
TRPC4	GK rat	Wistar rat	Endothelium-denuded caudal artery smooth muscle strips	Cirazoline- or cyclopiazonic acid-induced Ca^2+^ influx was decreased in diabetic rat	TRPC4 mRNA was not detected in Wistar rats, but it was detectable in GK rats	TRPC4 protein was not detected in Wistar rats, but it was barely detectable in GK rats	Mita et al. (2010)
TRPC5	GK rat	WKY rat	Cultured aortic vascular smooth muscle cell	Angiotensin-II-induced Ca^2+^ influx was enhanced in diabetic rat	No change	No change	Evans et al. (2009)
TRPC6	GK rat	WKY rat	Cultured aortic vascular smooth muscle cell	Angiotensin-II-induced Ca^2+^ influx was enhanced in diabetic rat	Decrease	Decrease	Evans et al. (2009)
TRPC6	Human diabetic	Human non-diabetic	Saphenous vein	Cyclopiazonic acid-induced Ca^2+^ influx was enhanced in diabetic patient	No change	Decrease	Chung et al. (2009)
TRPC6	GK rat	Wistar rat	Endothelium-denuded caudal artery smooth muscle strips	Cirazoline- or cyclopiazonic acid-induced Ca^2+^ influx was decreased in diabetic rat	–	Increase	Mita et al. (2010)
TRPM2	Streptozotocin (STZ)-treated lean Zucker (LZ) rats (Type I diabetes)	Lean Zucker rats	Pulmonary artery	Lung capillary filtration coefficient (Kf) was enhanced in diabetic rat. TRPM2 channel mediated increase in Kf.	–	Decrease	Lu et al. (2014)
TRPM2	High-fat diet (HFD)-fed mice C57BL/6J for 16 weeks.	Low-fat diet (LFD)-fed mice C57BL/6J for 16 weeks.	Mouse aortic endothelial cells and aortas	Preincubation of TRPM2 inhibitor N-(p-amylcinnamoyl) anthranilic acid (20 μM) or knockdown of TRPM2 alleviates obesity-associated impairment in insulin-evoked endothelium-dependent relaxations in obese mice	–	Increase	Sun et al. (2019)
TRPV1	Zucker diabetic fatty (ZDF) rat (Type II diabetes)	Genetic controls	Branch II and III mesenteric arteries. (A portion of the omental membrane, which frequently contains nerve trunks, was maintained)	Capsaicin-induced relaxation was similar in diabetic rat.	–	–	Pamarthi et al. (2002)
TRPV1	STZ -induced diabetic Sprague-Dawley rats	Sprague-Dawley rats	Epineurial arterioles of the sciatic nerve	Capsaicin-induced constriction (10^–6^ M) was decreased in diabetic rat (10–12-week duration).	–	Decrease	Davidson et al. (2006)
TRPV1	STZ -induced diabetic Wistar rats	Wistar rats treated with the solvent for STZ	Medial meningeal artery (Meningeal blood flow)	Capsaicin-induced relaxation (10^–7^ M) was abolished in diabetic rat. Capsaicin-induced constriction (10^–5^ M) was similar in diabetic rat.	–	–	Dux et al. (2007)
TRPV1	db/db mice (Type 2 diabetes and obesity)	C57BLKS/J mice	Mean arterial blood pressure (MAP) Aortic tissue	Capsaicin-induced increases in MAP was attenuated in diabetic mouse.	–	Decrease	Ohanyan et al. (2011)
TRPV1	db/db mice	C57BLKS/J mice	Coronary microvessel Myocardial blood flow (MBF)	Capsaicin-induced increases in MBF and capsaicin-mediated relaxation in coronary microvessels were attenuated in diabetic mouse.	–	–	Guarini et al. (2012)
TRPV1	db/db mice	C57BLKS/J mice	Thoracic aortas and mesenteric arteries	Dietary capsaicin improves the endothelium-dependent relaxation in diabetic mouse compared to db/db mice given a normal diet.	–	Decrease	Sun et al. (2013)
TRPV1	STZ -induced diabetic Sprague-Dawley rats	Sprague-Dawley rats	Third branch of the superior mesenteric artery	Capsaicin-induced relaxation was decreased in diabetic rat.	–	Decrease	Zhang et al. (2015)
TRPV1	db/db mice	C57BLKS/J mice	Coronary arterioles Coronary blood flow (CBF)	H_2_O_2_ had little potentiating effect on capsaicin-induced CBF responses or capsaicin-mediated coronary vasodilation in db/db and TRPV1 knockout mice.	–	–	DelloStritto et al. (2016)
TRPV1	Human diabetic (Type 1 diabetes)	Human non-diabetic	Cutaneous vascular conductance (CVC) in the forearm	CVC was decreased in diabetic patients in response to local heating early peak.	–	–	Marche et al. (2017)
TRPV1	High-fat/high-cholesterol diet- induced obese male Ossabaw miniature swine for 24 weeks.	Lean male Ossabaw miniature swine for 24 weeks.	Coronary arteries	Capsaicin-induced relaxation was impaired in obese pigs.	Increase	Decrease	Bratz et al. (2008)
TRPV1	HFD-fed Sprague-Dawley rats for 20–24 weeks.	Normal diet-fed Sprague-Dawley rats for 20–24 weeks.	Small mesenteric arteries (third-order)	Capsaicin (10 μM) significantly increased the amplitude of nerve-mediated contraction induced by 10 Hz stimulation, with a greater effect in control than obese animals.	–	–	Haddock and Hill (2011)
TRPV1	HFD-fed mice C57BL6/129SVJ for 12 weeks.	Normal diet-fed mice C57BL6/129SVJ for 12 weeks.	Aorta	Vascular hypertrophy was observed in HFD-fed wild-type but not HFD-fed TRPV1 knockout mice.	–	–	Marshall et al. (2013)
TRPV1	Obese Zucker (OZ) rats	LZ rats	Resistance mesenteric arteries	Capsaicin-induced relaxation was decreased in OZ rats	–	No change	Lobato et al. (2013)
TRPV1	High-fat, high-sucrose (HFHS) diet-induced obese Sprague-Dawley rats for 20 weeks.	Regular diet-fed Sprague-Dawley rats for 20 weeks.	Meningeal blood flow	Capsaicin-induced increased meningeal blood flow (100 nM) was greater in obese rat. Capsaicin-induced decreased meningeal blood flow (10 μM) was greater in obese rat.	–	–	Marics et al. (2017)
TRPV4	STZ -induced diabetic Sprague-Dawley rats	Sprague-Dawley rats	Third or fourth branches of rat mesenteric artery	TRPV4-K_*Ca*_2.3-mediated relaxation were impaired in diabetic rats	–	Decrease	Ma et al. (2013)
TRPV4	STZ -induced diabetic Sprague-Dawley rats	Sprague-Dawley rats	Retinal arteriole	–	Decrease	Decrease	Monaghan et al. (2015)
TRPV4	db/db mice and STZ -induced diabetic C57BLKS/J mice	C57BLKS/J mice	Aortas	–	Decrease	Decrease	Gao et al. (2020)
TRPV4	HFD-fed mice C57BL/6J. The diets initiated at age 5 weeks and continued at age 6 months.	LFD-fed mice C57BL/6J. The diets initiated at age 5 weeks and continued at age 6 months.	Third-order mesenteric arteries	Vasodilatory responses to GSK1016970A (TRPV4 agonist) in resistance mesenteric arteries were similar between the LFD- and HFD-fed mice.	–	–	Greenstein et al. (2020)
TRPV4	HFD-fed mice C57BL/6J for 14 weeks. Obese individuals.	LFD-fed mice C57BL/6J for 14 weeks. Non-obese individuals.	Resistance mesenteric arteries from mice. Splenius/temporalis muscle arteries from human.	Vasodilatory response to GSK1016970A was impaired in HFD mice. Vasodilatory response to GSK1016970A was markedly reduced in the arteries from obese individuals.	–	–	Ottolini et al. (2020)

### The Role of TRPM2 in the Vasculature Under Diabetic and Obese Conditions

TRPM2 is activated by H_2_O_2_ (Hara et al., 2002), adenosine 5′-diphosphoribose (ADP-ribose) (Heiner et al., 2003; Yu et al., 2017), nicotinic acid-adenine dinucleotide phosphate (NAADP) (Beck et al., 2006), Ca^2+^ (McHugh et al., 2003), and temperature (35–47°C) (Togashi et al., 2006; Kashio et al., 2012; Kashio and Tominaga, 2017), while adenosine monophosphate (AMP) (Beck et al., 2006; Lange et al., 2008) and acidic pH are negative regulators (Du et al., 2009; Starkus et al., 2010). This channel is expressed in VSMC (Yang et al., 2006) and vascular endothelial cells (Hecquet et al., 2008), and it is permeable to Ca^2+^, Na^+^ (Perraud et al., 2001; Sano et al., 2001; Kraft et al., 2004), and K^+^ (Sano et al., 2001). Moreover, physiological splice variants of TRPM2, including full-length TRPM2 (TRPM2-L) and a short splice variant (TRPM2-S), have been identified in endothelial cells (Hecquet and Malik, 2009; Hecquet et al., 2014) and VSMC (Yang et al., 2006).

TRPM2 is involved in endothelial permeability, as demonstrated by H_2_O_2_-induced Ca^2+^ influx *via* TRPM2 channels that results in endothelial hyperpermeability (Hecquet et al., 2008). Moreover, H_2_O_2_ activates TRPM2 to induce excessive Ca^2+^ influx, resulting in Ca^2+^ overload and consequently, cell death in vascular endothelial cells (Sun et al., 2012). Furthermore, ROS overproduction activates TRPM2 channels, leading to Ca^2+^ influx through TRPM2, which induces VSMC migration and proliferation that contributes to neointimal hyperplasia (Ru et al., 2015).

**FIGURE 2:**
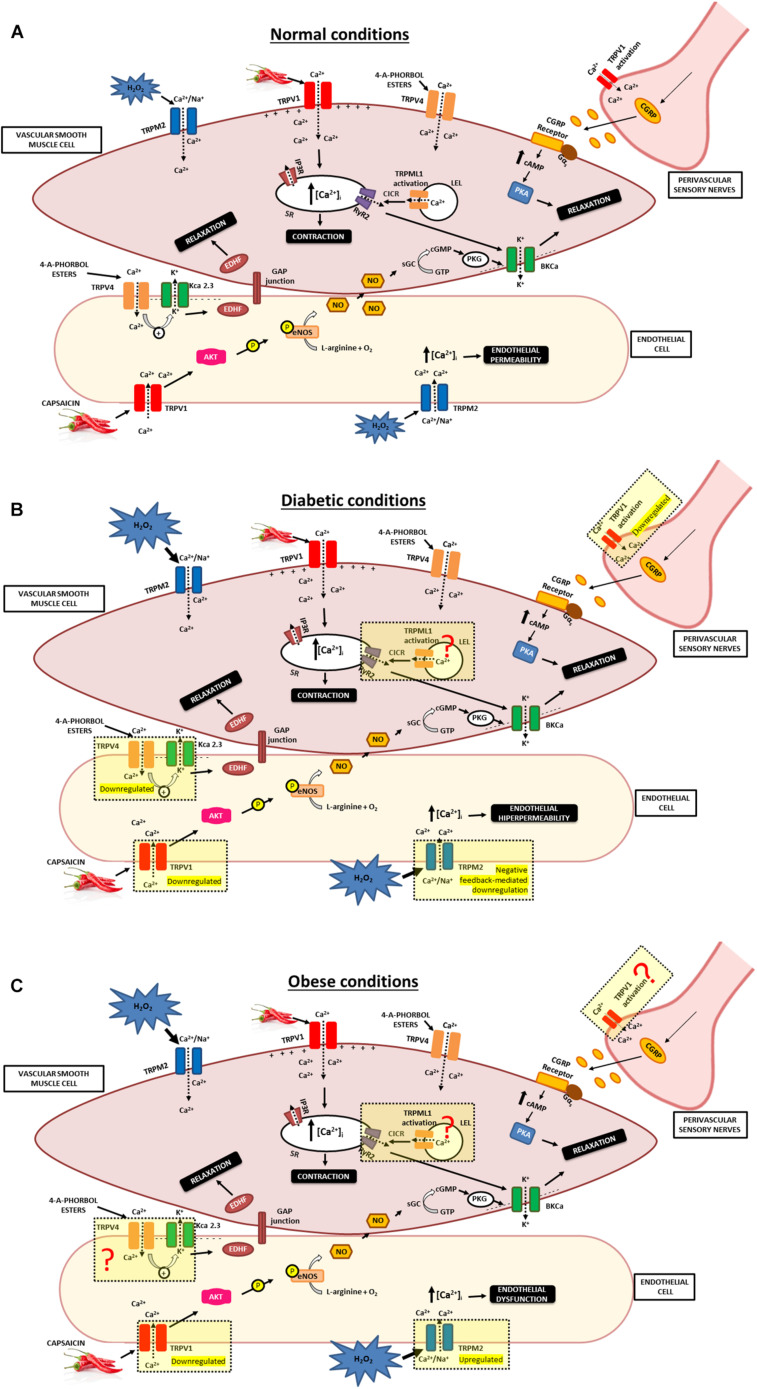
Involvement of TRPs in vascular responses in normal, diabetic and obese conditions. **(A)** The figure shows the possible mechanisms that can explain vasodilator influences of TRPV1, TRPV4, TRPM2, and TRPML1 channel present in the vasculature. TRPV1 channel activation causes release of the CGRP from sensory nerves. CGRP binds to CGRP receptor, inducing augmented levels of cAMP that activates PKA and promotes relaxation of VSMC. TRPV1 activation in endothelial cells promotes Ca^2+^ influx and phosphorylation of eNOS and induces NO production. NO active the soluble guanylyl cyclase, that catalyzes the conversion of GTP to cGMP and active the PKG. The NO/cGMP/PKG activates BK_*Ca*_ that leads to smooth muscle relaxation. Additionally, specific interaction of TRPV4 with K_*Ca*_2.3 in endothelial cells promote vasodilation, likely *via* an EDHF pathway. Moreover, H_2_O_2_-induced Ca^2+^ influx *via* TRPM2 channels in endothelial cells results in endothelial permeability. TRPML1 is closely associated with RyR2. TRPML1 activation provokes Ca^2+^ signals from a LELs, which can subsequently be augmented by CICR from the SR *via* RyR2 to induce Ca^2+^ sparks, leading to BK_*Ca*_ channel activity that result in membrane hyperpolarization, VSMC relaxation. **(B)** The figure shows the possible alterations in TRPV1, TRPV4, and TRPM2 channel under diabetic conditions. Diabetic conditions promote reduction in the capsaicin-evoked release of CGRP and decrease in the density of perivascular TRPV1. Moreover, a high level of glucose reduces TRPV1 expression and PKA phosphorylation in endothelial cells. Additionality, hyperglycemia is a crucial factor for the diminished TRPV4 expression and impairs the endothelium-dependent vasodilatation. Also, increased oxidative stress activates the TRPM2 channel and results in endothelial hyperpermeability. Besides that, overexposure of superoxide promoted a TRPM2 channel negative feedback-mediated downregulation. Further studies are needed to clarify whether TRPML1 activity and/or expression are altered in the vasculature during diabetes. **(C)** The figure shows the possible alterations in TRPV1 and TRPM2 channel under obese conditions. Impaired capsaicin-induced vasodilation in arteries is associated with reduced expression of TRPV1 protein and cation influx into endothelial cells under obese conditions. Increased oxidative stress present in obesity are modulating the TRPM2 channel, leading elevated activity of this channel. Further studies are needed to elucidate whether TRPV4 and TRPML1 activity and/or expression are altered in the vasculature during obesity. TRPV1, Transient receptor potential of vanilloid type 1; TRPV4, Transient receptor potential of vanilloid type 4; TRPM2, Transient receptor potential of melastatin type 2; TRPML1, Transient receptor potential of mucolipin type 1; VSMC, vascular smooth muscle cells; NO, nitric oxide; EDHF, endothelium-derived hyperpolarizing factor; eNOS, endothelial nitric oxide synthase; BK_*Ca*_, large-conductance Ca^2+^-activated K^+^ channel; K_*Ca*_2.3, small-conductance Ca^2+^-sensitive K^+^ channels (SK_*Ca*_) isoform. PKG, Protein Kinase G; cGMP, cyclic guanosine 3′,5′-monophosphate; GTP, guanosine 5′-triphosphate; SR, sarcoplasmic reticulum; IP_3_R, Inositol 1,4,5-trisphosphate receptor; RyR2, type 2 ryanodine receptors; cAMP, Cyclic adenosine monophosphate; PKA, protein kinase A; CGRP, Calcitonin gene-related peptide; CGRP receptor, Calcitonin gene-related peptide receptor; CICR, calcium-induced calcium release; LELs, late endosomes and lysosomes; H_2_O_2_, Hydrogen peroxide.

There are only a few studies that demonstrate changes in TRPM2 channel expression and/or function associated with diabetes and obesity. In pulmonary arteries from streptozotocin (STZ)-treated hyperglycemic lean Zucker (LZ) rats (type I diabetic), the TRPM2-L channel isoform was decreased when compared to controls. Contrarily, vascular superoxide levels, NADPH oxidase (NOX) activity and lung capillary filtration coefficient (Kf) are higher in STZ-treated LZ rats. Interestingly, inhibition of TRPM2 channel diminished lung Kf in diabetic rats but did not affect the Kf in control animals. The authors of this study proposed that in hyperglycemic rats, increased oxidative stress activates the TRPM2 channel and elevates pulmonary endothelial Kf. The decreased TRPM2-L expression through chronic hyperglycemia may be due to overexposure of superoxide and a subsequent negative feedback-mediated downregulation. This enhanced the TRPM2 activation-mediated increase in Kf that can contribute to the elevated susceptibility to lung complications observed in individuals with type I diabetes. Taken together, additional studies are needed to determine the pulmonary TRPM2 channel sensitivity in control and diabetic animal models by using electrophysiological and pharmacological tools (Lu et al., 2014; Figures 2A,B).

A study developed by Sun et al. (2019) demonstrated that TRPM2 expression significantly increased in both primary mouse aortic endothelial cells and aortic endothelium from high-fat diet (HFD, 60 kcal% fat)-fed mice. In addition, preincubation of the TRPM2 inhibitor *N*-(p-amylcinnamoyl) anthranilic acid (20 μM), reduced the impaired insulin-induced relaxation in aortas from HFD-fed mice. Similarly, knockdown of TRPM2 alleviated endothelial insulin resistance and improved endothelium-dependent vasodilatation in obese mice. The authors proposed that free fatty acid-induced H_2_O_2_ activation of TRPM2, thereby aggravating endothelial insulin resistance. Therefore, downregulation or pharmacological inhibition of TRPM2 channels may contribute to treatment of endothelial dysfunction associated with the oxidative stress state (Sun et al., 2019; Figures 2A,C). Both of these studies indicated that increased oxidative stress, present in diabetes and obesity, are modulating the TRPM2 channel (Table 1), leading to elevated channel activity. In this context, the decreased vascular TRPM2-L expression in the lung from diabetic animals, as shown by Lu et al., is due to negative feedback.

### The Role of TRPV1 in the Vasculature Under Diabetic and Obese Conditions

TRPV1 channels are expressed in endothelial cells (Yang et al., 2010), VSMC (Kark et al., 2008), perivascular sensory nerves (Zygmunt et al., 1999; Breyne and Vanheel, 2006), and pericytes (Tóth et al., 2005). TRPV1 channels are present in blood vessels, such as epineural arterioles (Davidson et al., 2006), aorta (Ohanyan et al., 2011; Sun et al., 2013), mesenteric (Sun et al., 2013; Zhang et al., 2015), and coronary arteries (Bratz et al., 2008). These channels are activated by multiple stimuli, including heat (∼42–51°C) (Tominaga et al., 1998; Cesare et al., 1999), anandamide (Zygmunt et al., 1999), and exogenous agonists, such as capsaicin and resiniferatoxin (Caterina et al., 1997), as well as low pH that acts as a sensitizing agent (Tominaga et al., 1998; Cesare et al., 1999). TRPV1 is a non-selective cation channel, which is permeable to K^+^, Na^+^, Ca^2+^, and Mg^2+^ (Caterina et al., 1997).

Activation of TRPV1 by capsaicin promotes the release of neurotransmitters, such as calcitonin gene-related peptide (CGRP) (Zygmunt et al., 1999; Wang et al., 2006) from capsaicin-sensitive nerves, in addition to NO from endothelial cells (Yang et al., 2010; Ching et al., 2011), which can diffuse to adjacent VSMC and cause relaxation. In smooth muscle cells from skeletal muscle arterioles obtained from the rat and mice, TRPV1 stimulation causes an increase in intracellular Ca^2+^ concentration, resulting in vasoconstriction (Czikora et al., 2012). Therefore, the activation of TRPV1 may induce different effects on the vasculature (vasoconstriction, vasodilation, or no effect), which can be unique to each vascular bed. For example, arteries with sensory neuron innervation and without vascular TRPV1 expression are expected to dilate in response to TRPV1 activation. However, arteries with elevated smooth muscle TRPV1 expression and without apparent sensory neuronal innervation constrict in response to the same TRPV1 stimulation (Kark et al., 2008; Tóth et al., 2014). Moreover, TRPV1 activation by capsaicin induced concentration-dependent biphasic effects, where a low concentration capsaicin evoked dilation, while a higher concentration resulted in vasoconstriction of the dural vessels (Dux et al., 2003) and skeletal (musculus gracilis) muscle arterioles (Kark et al., 2008).

Abundant evidence supports the hypothesis that altered TRPV1 expression and/or function is associated with vascular dysfunction in diabetes and obesity. The TRPV1 is the most studied TRP channel in the vasculature, under these metabolic conditions. In humans, a study by Marche et al. (2017) evaluated cutaneous vascular conductance (CVC) in response to heat by using a skin-heating probe, heated to 44 °C to assess heat-induced vasodilation. The local heat-induced early peak is mediated through TRPV1 channels, located on sensory nerves. Therefore, the significantly diminished peak response to local thermal hyperemia could suggest reduced activity of the TRPV1 channels at the skin level in type 1 diabetic patients compared to control subjects. This study indicated that the microvascular response triggered by TRPV1 channels is reduced in type 1 diabetic patients (Marche et al., 2017).

Zhang et al. (2015) investigated the pharmacological effects of capsaicin on mesenteric arteries of STZ-induced diabetic Sprague-Dawley rats. Capsaicin-induced vasodilation was impaired in the mesenteric arteries of diabetic rats. As well, TRPV1 expression was reduced in the diabetic preparation when compared to the control group. The authors indicated that the attenuated expression of CGRP and TRPV1 contribute to the weakened capsaicin-mediated dilation in diabetic mesenteric arteries (Zhang et al., 2015). In line with previous studies, capsaicin-induced relaxation in resistance mesenteric arteries was markedly decreased in obese Zucker (OZ; genetic model of obesity) rats compared with LZ rats. However, TRPV1 receptor protein expression was similar between LZ and OZ rats. The authors suggest that the weakened vascular effect to anandamide in arteries from this obese model can involve reduced activation of C-fiber nerve endings, and this may collaborate to the vascular dysfunction observed in OZ rats (Lobato et al., 2013). However, one concern about this model is due the mutation of the *fa* gene (cause of obesity in OZ rats) is not common among humans.

In addition, the study by Dux et al. (2007) evaluated the TRPV1 receptor-mediated neurogenic sensory vasodilation in diabetic rats. In control and insulin-treated diabetic animals, capsaicin (10^–7^ M) induced increases in meningeal blood flow, but in 6-week STZ-induced diabetic rats, capsaicin promoted decreases in the blood flow. In contrast, capsaicin at a higher concentration (10^–5^ M) caused vasoconstriction, which is a non-neurogenic response and was similar in control and diabetic animals. The authors demonstrated a reduction in the capsaicin-evoked release of CGRP and decrease in the density of perivascular and stromal TRPV1-immunoreactive nerve fibers of the dura mater from diabetic rats, suggesting that insufficient vasodilator function of meningeal sensory nerves may contribute to the higher incidence of headaches in diabetics due to perturbation of tissue homeostasis that could induce additional activation and/or sensitization of meningeal nociceptors (Dux et al., 2007). Further studies are needed to determine if this hypothesis can be supported. It is pertinent to highlight the fact that diabetic rats treated with insulin restored the vasodilatory response and the capsaicin-evoked release of CGRP, indicating that impairments observed in diabetic animals can be attributed to the diabetic condition induced by STZ and not to a toxic action of this drug. Moreover, it is important to note, that the current evidences demonstrate that TRPV1 channels expression and/or activity in perivascular sensory nerves are reduced under these conditions.

In an opposite way, in model of obesity, topical administration of capsaicin (100 nM) to the dura mater promoted enhanced meningeal blood flow in high-fat high-sucrose (HFHS) diet-fed Sprague–Dawley rats (diets started at 6 weeks of age and continued for 20 weeks; 45% of total calories as fat) compared to regular diet-fed rats. However, administration of capsaicin at 10 μM induced a greater reduction in meningeal blood flow in obese animals compared to controls. In this way, dural application of capsaicin resulted in significantly higher vasodilator and vasoconstrictor responses in obese animals compared to controls. Moreover, this obesity animal model was characterized by an increase in CGRP release in response to both concentrations of capsaicin administered, suggesting a greater TRPV1-mediated CGRP release from meningeal afferent nerves likely due to a sensitization of the TRPV1 receptor. This sensitization may be a consequence of the increase in proinflammatory cytokines and levels of oxidative stress. Changes in TRPV1-mediated vascular reactions and CGRP release, may be related to the enhanced headache susceptibility of obese individuals (Marics et al., 2017). Moreover, Dux et al. (2007) and Marics et al. (2017) demonstrated divergent results on TRPV1 receptor-mediated neurogenic sensory vasodilation between diabetic and obese conditions, indicating that different mechanisms can contribute to modulation of the TRPV1 channels in each disease.

Guarini et al. (2012) showed that capsaicin-mediated increases in myocardial blood flow (MBF), using myocardial contrast echocardiography, were reduced in db/db mice, a model of type II diabetes, and obesity. Similarly, relaxation promoted by capsaicin was attenuated in coronary microvessels from diabetic mice. Interestingly, myocardial pH was more acidic in diabetic mice than control mice and pH-mediated relaxation was attenuated in coronary microvessels from TRPV1^(–/–)^ and db/db mice. The authors speculated that TRPV1 channels directly regulate MBF and impairment of TRPV1 channels could contribute to vascular dysfunction that is typically observed in diabetes. As previously described, lowering pH is a stimulus for TRPV1 activation. The study by Guarini et al. (2012) demonstrates a possible desensitization of TRPV1 in situations of prolonged acidic environment exposure. Further investigation into prolonged acidic environment on TRPV1 desensitization is necessary.

A follow-up study by this group reported that acute H_2_O_2_ exposure potentiated capsaicin-mediated coronary blood flow (CBF), using the same methodology that was described by Guarini et al. (2012), responses and capsaicin-induced dilation of coronary microvessels in control mice, but H_2_O_2_ had little potentiating effect on capsaicin-mediated responses in db/db and TRPV1 knockout mice. However, after excessive H_2_O_2_ exposure, CBF and microvessel responses in the control mice resembled those of the attenuated responses seen in TRPV1 knockout and db/db mice. The author indicated that H_2_O_2_-induced increases in CBF are promoted, in part, by TRPV1 channels. Moreover, prolonged H_2_O_2_ exposure disrupts TRPV1-dependent coronary vascular signaling, which can cause in-tissue perfusion impairments observed in diabetes (DelloStritto et al., 2016).

Sun et al. (2013) demonstrated that cultured endothelial cells that are exposed to a high level of glucose (30 mmol/L), reduced TRPV1 expression and protein kinase A (PKA) phosphorylation compared with control cells and that these effects were reversed by the administration of capsaicin (1 μmol/L). Similarly, in the aorta and mesenteric arteries from db/db mice, TRPV1 expression and PKA phosphorylation were decreased, but uncoupling protein 2 (UCP2) level was significantly higher when compared to wild type mice. After dietary administration of 0.01% capsaicin for 14 weeks, TRPV1 activation induced PKA phosphorylation and elevated the expression level of UCP2 in diabetic mice. Moreover, capsaicin ameliorated vascular oxidative stress and increased NO levels in db/db mice. The authors concluded that TRPV1 activation by capsaicin might attenuate hyperglycemia-induced endothelial dysfunction through a mechanism involving the PKA and UCP2-mediated antioxidant effect (Sun et al., 2013). If this conclusion is accurate, then it would indicate a possible target for future research on chronic treatment with TRPV1 agonists in the diabetic and obesity conditions, evaluating whether these agonists could attenuate or prevent vascular dysfunction. In addition, these studies demonstrate new possibilities of capsaicin-rich dietary recommendations for complementary assistance in the treatment of diabetic patients.

Similarly, Bratz et al. (2008) demonstrated impaired capsaicin-induced vasodilation in coronary arteries from obese Ossabaw swine (diets were provided for 24 weeks; 46% of total kcal from fat) associated with reduced expression of TRPV1 protein and cation influx into endothelial cells. On the other hand, TRPV1 channel mRNA expression was increased in obese swine compared with lean controls. The authors concluded that TRPV1 channel signaling is diminished in metabolic syndrome and this disrupted pathway can contribute to the endothelial dysfunction and the development of coronary artery disease (Bratz et al., 2008). These findings support the notion that decreased expression of TRPV1 channel and Ca^2+^ influx into endothelial cells promote insufficient vasodilator response, collaborating to the endothelial dysfunction related to diabetic and obesity conditions.

Together, these studies support a model in which activation of TRPV1 channels from endothelial cells and perivascular sensory nerves cause vasodilation. This mechanism may be disrupted during diabetes and obesity, contributing to vascular dysfunction associated with these conditions, resulting in higher incidence of headaches, coronary disease, and tissue perfusion impairment.

However, Pamarthi et al. (2002) demonstrated that capsaicin-induced concentration-dependent relaxation of branch II and III mesenteric arteries and CGRP nerve density was similar in the Zucker diabetic fatty (ZDF) rat, a model of type II diabetes, and genetic controls. ZDF rats exhibit obesity, severe hyperglycemia, an early hyperinsulinemia and dyslipidemia. Moreover, the obesity is promoted by the *fa* leptin receptor mutation (Pamarthi et al., 2002), but, as described before, this is not common cause of obesity among humans.

In contrast, Davidson et al. (2006) reported that capsaicin induced a concentration-dependent vasoconstriction of epineural arterioles of the sciatic nerve from Sprague-Dawley rats, concluding that vasoconstriction was likely due to the release of neuropeptide Y (NPY) contained in nerves that innervate these arterioles. However, vasoconstriction to capsaicin was significantly decreased in long-term diabetic rats. This altered response was correlated with the reduced expression of TRPV1 in epineural arterioles in diabetic rats (Davidson et al., 2006). Moreover, the present evidence shows that TRPV1 channels expression and/or activity in sensory nerves that innervate these arterioles are decreased under diabetic condition. Overall, these findings are in accordance with findings reported by Haddock and Hill (2011). In an animal model of obesity, capsaicin (10 μM) promoted a significant increase in nerve-mediated vasoconstriction induced by a 10 Hz stimulation in small mesenteric arteries from groups fed a high-fat (diets started at 6 weeks of age and were provided for 20–24 weeks; containing 43% of total calories as fat) and normal diet, although the effect was greater in control rats (Haddock and Hill, 2011). From the results, it is clear that common factors between obesity and diabetes can modulate TRPV1 channel, leading to the reduced vasoconstriction. Additional studies to investigate which specific mechanisms collaborate to TRP channels modulation in each disease are necessary.

A study by Ohanyan et al. (2011) showed that capsaicin caused an increase in mean arterial blood pressure (MAP) in mice, but the increase MAP was attenuated in the db/db mice. In addition, mice were given the ganglion blocker, hexamethonium, to evaluate the primary actions of capsaicin and to eliminate reflex adjustments. Furthermore, this diminished capsaicin-induced pressor response was correlated with reduced aortic TRPV1 protein expression in db/db mice. Moreover, cultured bovine aortic endothelial cells exposed to capsaicin augmented endothelin production and endothelin A (ET_*A*_) receptor inhibition reduced the capsaicin-mediated rises in MAP. Based on these findings, the authors indicated that TRPV1 channels are involved in the regulation of vascular reactivity and systemic pressure through production of endothelin, resulting in activation of vascular ET_*A*_ receptors. Therefore, a decrease in vascular TRPV1 channel expression may contribute to vascular dysfunction in diabetes. The authors suggest that this reduced TRPV1 channels could promote sensitization of vasoconstrictor pathways and reduced functional hyperemia present in diabetic patients (Ohanyan et al., 2011). A limitation of this study was the use of conductance vessels instead of resistance vessels in order to evaluate TRPV1 protein expression. Moreover, further studies should evaluate if substance P and NPY can participate in the capsaicin-mediated pressor response.

Marshall and colleagues revealed that hypertension and vascular hypertrophy were observed in HFD-fed wild-type (diets for 12 weeks from 3 weeks of age; 35% fat from lard) but not HFD-fed TRPV1 knockout mice, indicating that the onset of vascular remodeling may have an association between TRPV1 and obesity-induced high blood pressure. Moreover, constrictor and dilator responses to phenylephrine, CGRP, and the endothelium-dependent carbachol remained intact, suggesting little vascular dysfunction in the mesenteric resistance artery in this obese model. Interestingly, the authors provided evidence that TRPV1 knockout mice were protected from obesity-induced hypertension and vascular hypertrophy (Marshall et al., 2013; Table 1). However, it is important to note that these results differ from studies that have linked decreased TRPV1 expression or/and function with a worsened phenotype. Moreover, there is no significant alteration on the mean arterial pressure in TRPV1 knockout mice related to wild-type mice under normal diet. This implies that altered TRPV1 activity can be associated with a compensatory response that counteracts the hypertension in this model of obesity. The HFD-wild-type mice show low-grade inflammation, reducing glucose tolerance and raised levels of adipokine that could be involved with modulation of this channel. Furthermore, it cannot be ruled out that the different influences of TRPV1 channels on the vasculature depend on the tested diabetic or obese animal model. Thus, additional research is needed to confirm these observations.

Collectively, these findings reveal the downregulated TRPV1 channel expression is related to the diabetic condition (Figures 2A,B). In obese animal models, these studies demonstrated alterations in TRPV1 channel expression and/or function, suggesting a role of TRPV1 in obese conditions (Figures 2A,C). Nevertheless, the data obtained from these studies are divergent, which can be justified by the use of different obesity animal models, observed by distinct diet compositions, durations and age of onset of diet intervention, which can result in different metabolic profiles and severity of obesity. In addition to the different models, different vascular beds were utilized which confound the conclusion’s coalescence. Overall, these findings demonstrate that mainly TRPV1 channels in endothelial cells and perivascular sensory nerves are altered under diabetic and obese conditions.

### The Role of TRPV4 in the Vasculature Under Diabetic and Obese Conditions

TRPV4 is expressed in the aorta (Gao et al., 2020), mesenteric (Ma et al., 2013), carotid (Hartmannsgruber et al., 2007), pulmonary (Martin et al., 2012), cerebral basilar (Han et al., 2018), and renal (Soni et al., 2017) arteries, among others, and it can be present in both VSMC (Martin et al., 2012; Soni et al., 2017) and the endothelium (Marrelli et al., 2007; Ma et al., 2013; Han et al., 2018). A broad range of stimuli can lead to TRPV4 activation, including heat (>27°C) (Güler et al., 2002; Watanabe et al., 2002b), hypoosmotic conditions (Liedtke et al., 2000; Strotmann et al., 2000; Alessandri-Haber et al., 2003), low pH and citrate (Suzuki et al., 2003), 5,6- epoxyeicosatrienoic acid (Watanabe et al., 2003), and 4-α-phorbol esters (Watanabe et al., 2002a, b). TRPV4 is a nonselective cation channel, permeable to Ca^2+^, Mg^2+^ and K+ (Voets et al., 2002), and it exhibits moderate permeability to Ca^2+^ (P_*Ca*_/P_*Na*_∼6) (Strotmann et al., 2000; Voets et al., 2002; Watanabe et al., 2002a).

Moreover, there is evidence that TRPV4-mediated stimulation of intermediate-conductance Ca^2+^-sensitive K^+^ channels (IK_*Ca*_) and/or small-conductance Ca^2+^-sensitive K^+^ channels (SK_*Ca*_) channels can promote vasodilation, likely *via* an EDHF pathway (Zhang et al., 2013; Han et al., 2018). For example, there is a functional interaction between TRPV4 and the K_*Ca*_2.3, SK_*Ca*_ isoform, in endothelial cells (Sonkusare et al., 2012). This association plays a key role in smooth muscle hyperpolarization and relaxation (Ma et al., 2013; Lu et al., 2017; Huang et al., 2019). Additionally, Ca^2+^ entry through endothelial TRPV4 channels can trigger NO-dependent vasodilation (Köhler et al., 2006; Marziano et al., 2017).

TRPV4 channel expression appears to be altered in diabetic conditions and has a significant impact on the regulation of vascular tone. Ma et al. (2013) were the first to demonstrate evidence of the physical interaction between TRPV4 and K_*Ca*_2.3 in endothelial cells from the rat mesenteric artery. The expression levels of TRPV4 and K_*Ca*_2.3 were reduced and TRPV4-K_*Ca*_2.3-mediated relaxation was impaired in STZ-induced diabetic rats. The authors proposed that the reduced TRPV4-K_*Ca*_2.3 signaling could be an underlying mechanism for EDHF dysfunction in diabetic rats (Ma et al., 2013).

Similarly, protein expression of endothelial TRPV4 in the retinal vasculature was reduced in STZ-induced diabetic rats compared with age-matched controls. The authors speculated that TRPV4 channel downregulation may contribute to impaired endothelium-dependent relaxation and retinopathy (Monaghan et al., 2015). Similarly, in db/db and STZ -induced diabetic C57BLKS/J mice, mRNA and protein levels of TRPV4 were significantly decreased in aortas, indicating that hyperglycemia is a crucial factor for the diminished TRPV4 expression, and impairs the endothelium-dependent vasodilation observed in diabetic mice (Gao et al., 2020).

A recent report demonstrated that diet-induced obesity (diets started at 6 weeks of age and continued until at 20 weeks; 60% of total kcal from fat) is associated with impaired Ca^2+^ influx through TRPV4 channels and vasodilation induced by muscarinic stimulation and GSK1016970A (TRPV4 agonist) in resistance mesenteric arteries from mice. Increased activities of inducible nitric oxide synthase (iNOS) and NOX1 enzymes at myoendothelial projections (MEPs) in obese mice produced higher levels of NO and superoxide radicals, resulting in augmented local peroxynitrite formation and subsequent oxidation of the regulatory protein AKAP150, to impair AKAP150-TRPV4 channel signaling at MEPs. Similarly, vasodilation was also weakened in the splenius/temporalis muscle arteries and peroxynitrite causes the impairment of endothelial TRPV4 channel activity in arteries from obese patients. Inhibition of iNOS or lowered peroxynitrite levels may be a strategy to restore TRPV4 channel activity and vasodilation in the obese condition (Ottolini et al., 2020).

In contrast, a HFD mouse model of obesity (diets initiated at age 5 weeks and continued until at age 6 months; 60% of total calories from fat), the vasodilator function induced by muscarinic stimulation of the endothelium and the underlying endothelial TRPV4 channel–mediated Ca^2+^ sparklet entry was not affected in resistance mesenteric arteries from obese mice. Vasodilator responses to GSK1016970A were similar between the mice receiving LFD and HFD. Similarly, there was no change in diameter of the pressure constricted arteries from either HFD or LFD mice in response to TRPV4 inhibition (HC067047). However, these obese animals exhibit Ca^2+^ spark–BK_*Ca*_ dysfunction that can be associated to development of obesity-related hypertension (Greenstein et al., 2020; Table 1). These studies by Ottolini et al. (2020) and Greenstein et al. (2020) have performed similar approaches, using third-order mesenteric arteries pressurized to 80 mmHg, and internal diameter was recorded in response to numerous treatments. As an alternative to these contradicting findings, TRPV4 can play a compensatory role aimed at restoring blood pressure in the study by Greenstein et al. (2020) or additional variables such as the duration on diet, genetic drift and discrepancies in the microbiome, can be associated to the differences found in the TRPV4 channel activity.

Taken together, these reports reveal that the downregulated TRPV4 channel expression is related to impaired vasorelaxation in diabetes (Figures 2A,B). In animal models of obesity, studies demonstrated divergent results (Figures 2A,C), Ottolini et al. (2020) evidenced that reduced TRPV4 channels function can contribute to obesity-induced hypertension, while contrarily, a study by Greenstein et al. (2020) showed no alteration in TRPV4 expression and/or activity, therefore obesity had no influence on the endothelial muscarinic/TRPV4 vasodilator pathway. Moreover, these HFD mouse models of obesity have slight difference between duration of diets. Further studies are clearly needed to confirm these findings.

### The Potential Role of TRPML1 in the Vasculature Under Diabetic and Obese Conditions

TRPML is the most recently identified subfamily of TRP (Bargal et al., 2000; Bassi et al., 2000; Sun et al., 2000), consisting of three members, TRPML1, TRPML2, and TRPML3 (Venkatachalam et al., 2006; see review of Samanta et al., 2018). TRPML1 channels are broadly distributed, located in the lung, heart, skeletal muscle, placenta (Bassi et al., 2000), and VSMC (Thakore et al., 2020), among others. TRPML2 is expressed in gliomas (Morelli et al., 2016), lymphoid and myeloid tissues (Lindvall et al., 2005; Samie et al., 2009), and TRPML3 is most abundant in the cochlea, melanocytes in skin hair follicles (Xu et al., 2007), vomeronasal and olfactory receptor neurons (Castiglioni et al., 2011). Moreover, TRPML1 is the only TRPML member present in smooth muscle cells from cerebral and mesenteric arteries (Thakore et al., 2020).

TRPML1 channels are mainly localized to the membranes of late endosomes and lysosomes (LELs) (Pryor et al., 2006; Vergarajauregui and Puertollano, 2006; Dong et al., 2008), and it is permeable to multiple ions including Ca^2+^, Na^+^, K^+^ (LaPlante et al., 2002), and Fe^2+^ (Dong et al., 2008). Moreover, this channel is transiently modulated by changes in cytosolic Ca^2+^ (LaPlante et al., 2002) and phosphatidylinositol 3,5-bisphosphate [PI(3,5)P2] (Dong et al., 2010). TRPML1 channels participate in some cell functions, including autophagy (Scotto Rosato et al., 2019), exocytosis (LaPlante et al., 2006; Samie et al., 2013), membrane trafficking (LaPlante et al., 2004) and H^+^ homeostasis (Soyombo et al., 2006).

Zhang et al. (2006) showed that lysosomes act as a crucial Ca^2+^ store and play a role in Ca^2+^ mobilization in coronary arterial smooth muscle cells and subsequently, vasoconstriction of coronary arteries. In this way, the lysosomal luminal concentration of Ca^2+^ is ∼0.5 mM, which is higher than cytosolic Ca^2+^ at ∼100 nM (Christensen et al., 2002). Additionally, NAADP can selectively provoke Ca^2+^ signals from a lysosome-related Ca^2+^ store alone, which can subsequently be augmented by calcium-induced calcium release (CICR) from the sarcoplasmic reticulum/endoplasmic reticulum *via* the ryanodine receptor (Kinnear et al., 2004). Moreover, TRPML1 can act as a NAADP-sensitive Ca^2+^ release channel and mediate Ca^2+^ release in lysosomes from the liver in rats (Zhang and Li, 2007) and from bovine coronary arterial muscle (Zhang et al., 2009).

A recent study by Thakore et al. demonstrated that TRPML1 is closely associated with type 2 ryanodine receptors (RyR2), inducing Ca^2+^ sparks in native arterial myocytes. Additionally, TRPML1 channels, acting upstream of RyR2s, were crucial in the spontaneous generation of Ca^2+^ sparks, leading to BK_*Ca*_ channel activity that resulted in membrane hyperpolarization, arterial myocyte relaxation, and vasodilation. Consequently, mice deficient in TRPML1 (*Mcoln1^–/–^*) resulted in excessive vasoconstriction and hypertension. The authors concluded that under physiological conditions, TRPML1 channels initiate Ca^2+^ sparks, thus diminishing myocyte contractility to regulate vascular resistance and blood pressure (Thakore et al., 2020). This work provides unpredicted results that support an unconventional role for TRPML1 channels in arterial smooth muscle cells and hypertension. In this way, we speculated that the TRPML1 channel could have a potential role in the vasculature under diabetic and obese conditions. Further studies are needed to clarify whether TRPML1 activity and/or expression are altered in the vasculature during cardiometabolic disorders, such as obesity and diabetes, so far lacking in the scientific literature.

## The Role of Perivascular Adipose Tissue (PVAT) and Reactive Oxygen Species (ROS) to the Vascular Dysfunction

Perivascular adipose tissue is in close proximity with the vasculature, and it surrounds most blood vessels, including aortic (Azul et al., 2020), coronary (Payne et al., 2009), brachial (Rittig et al., 2008) and mesenteric (Fésüs et al., 2007) arteries. PVAT is considered an active endocrine organ, producing and releasing many bioactive signaling molecules, such as: superoxide (Gao et al., 2006), hydrogen peroxide (Gao et al., 2007), tumor necrosis factor-α (TNF-α) (Virdis et al., 2015), leptin (Gálvez-Prieto et al., 2012), adiponectin (Meijer et al., 2013), visfatin (Wang et al., 2009), angiotensin (1–7) (Lee et al., 2009), and exosomes (Zhao et al., 2019). Upon secretion into the circulation, these molecules play an important role on vascular function, modulating the vasodilation by endothelium-independent and dependent pathways (Dubrovska et al., 2004; Salcedo et al., 2007; Yamawaki et al., 2009, 2010). In obesity and diabetes, PVAT dysfunction can induce vascular injury by mechanisms that include raised levels of pro-inflammatory cytokines, enhanced oxidative stress, pro-oxidant/antioxidant imbalance (Greenstein et al., 2009; Ketonen et al., 2010; Gil-Ortega et al., 2014; Azul et al., 2020), and a modification in the adipokine secretory profile (Saxton et al., 2019).

In addition, ROS are generated as a by-product of the cellular oxidative metabolism, and they are reactive molecules containing oxygen such as hydrogen peroxide, superoxide, and hydroxyl radical (Schieber and Chandel, 2014). In physiological levels, ROS play an important role in the regulation of numerous biological events, including proliferation (Arana et al., 2012), and angiogenesis (Wang et al., 2020) whereas excessive ROS (oxidative stress) are involved to several pathological conditions such as obesity (da Costa et al., 2017), and diabetes (Coughlan et al., 2009). As a result, oxidative stress can induce vascular dysfunction, leading reduced NO bioavailability (Cho et al., 2013), elevated peroxynitrite formation, eNOS uncoupling (Gamez-Mendez et al., 2015), and VSMC proliferation (Zhou et al., 2016).

Therefore, PVAT dysfunction and enhanced oxidative stress, present in diabetes and obesity, contribute to vascular damage (Greenstein et al., 2009; Ketonen et al., 2010; Gil-Ortega et al., 2014; Azul et al., 2020). Highlighting that PVAT dysfunction can be source of an abnormal generation of ROS (Ketonen et al., 2010; Azul et al., 2020). However, the literature is scarce to report the direct influences of products from PVAT on the TRP channels, despite oxidative stress modifying the expression and/or activity of the TRP channels. For example, increased oxidative stress promotes overactivation of TRPM2 channel in diabetes (Lu et al., 2014) and obesity (Sun et al., 2019). In contrast, peroxynitrite causes the impairment of endothelial TRPV4 channel activity by oxidation of the regulatory protein A-kinase anchoring protein 150 (AKAP150) (Ottolini et al., 2020).

In addition, levels of leptin are higher in obese individuals than in lean ones, leptin can induce hypertension by enhancing TRPM7 channel expression in the carotid body glomus cells and increasing TRPM7 activity (Shin et al., 2019). Moreover, leptin can stimulate TRPC channel, inducing vasoconstriction in endothelium-denuded pulmonary artery and thoracic aorta (Gomart et al., 2017). However, adipose-derived exosomes can reduce the pulmonary barrier hyperpermeability by inhibiting the TRPV4/Ca^2+^ pathway in HFD-induced obesity (Yu et al., 2020). As well, adiponectin can inhibit the expression of TRPV1 at the central terminals, modulating thermal sensitivity in physiological and neuropathic pain conditions (Sun et al., 2018). Consequently, biologically active compounds secreted by PVAT can modulate TRP channels. Furthermore, the secretory profile of PVAT is altered by obesity and diabetes, this may contribute to vascular dysfunction.

## Conclusion

**FIGURE 3:**
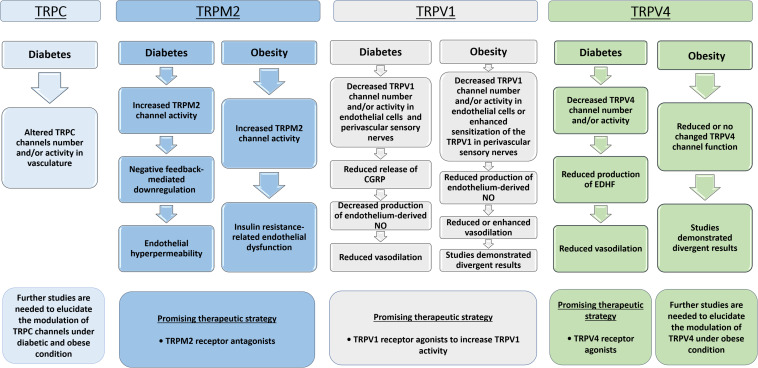
Flowchart showing the TRPs modulation in vascular responses under diabetic and obese conditions. TRPC, Transient receptor potential of canonical or classical; TRPV1, Transient receptor potential of vanilloid type 1; TRPV4, Transient receptor potential of vanilloid type 4; TRPM2, Transient receptor potential of melastatin type 2; NO, nitric oxide; EDHF, endothelium-derived hyperpolarizing factor; CGRP, Calcitonin gene-related peptide.

Robust evidence demonstrated that TRPC, TRPM and TRPV channels are involved in pathophysiological responses in the vasculature of animals with metabolic diseases (Figure 3). These disease mechanisms consist of altered expression or activation of TRP channels leading to impaired vasorelaxation, endothelial hyperpermeability, vascular hypertrophy or elevated contractility. In this context, TRP channels could be potential targets for the development of novel therapies to treat vascular dysfunction related to obesity and diabetes. However, additional investigations are necessary to completely elucidate the pathophysiological aspects of vascular TRP channels in obesity and diabetes. Furthermore, clinical researches are lacking in this area, so further clinical studies in this field are required.

However, there does exist a heterogeneity among the obese and diabetic animal models used in these studies. For instance, the severity of the obesity and the metabolic alterations can vary greatly between genetic versus diet-induced obesity. Moreover, there are differences in relation to the duration and the type of fat-diet consumed. In the same way, these studies demonstrated animal models of type 1 and 2 diabetes with different stages of diabetes. Nevertheless, it remains unclear whether the reported findings, in determined animal models can be attributed to the obese or diabetes state, regardless of the etiology.

## Author Contributions

RM drafted the manuscript. RW and DS contributed to conceptualization, review, and editing. All authors contributed to the article and approved the submitted version.

## Conflict of Interest

The authors declare that the research was conducted in the absence of any commercial or financial relationships that could be construed as a potential conflict of interest.
